# SNP-SIG 2013: from coding to non-coding - new approaches for genomic variant interpretation

**DOI:** 10.1186/1471-2164-15-S4-S1

**Published:** 2014-05-20

**Authors:** Yana Bromberg, Emidio Capriotti

**Affiliations:** 1 Department of Biochemistry and Microbiology; 2 Department of Genetics Rutgers University, New Brunswick, NJ, USA; 3 Division of Informatics, Department of Pathology; 4 Department of Biomedical Engineering University of Alabama at Birmingham, Birmingham, AL, USA

## Overview

The last few years have seen an explosion in genomic sequencing and, consistently, exponential growth in the number of known variants [1]. One of the variant repositories, **the dbSNP **database [2], currently contains over 62 million human single nucleotide polymorphisms (SNPs) and many other short genomic variants. These variants are interesting as both markers of evolution and for their phenotypic effects (e.g. characteristic traits and diseases). However, due to the high count of variants per-genome, an even larger number of potential variant set interactions, and relatively low levels of experimental annotation, variant interpretation is still severely limited.

In line with the continued strong interest of the computational biology community in genetic variation, the 3^rd ^SNP Special Interest Group (SNP-SIG) meeting [3,4] was held on July 19 at the ISMB/ECCB 2013 in Berlin (Germany). The meeting aimed to summarize the relevant (computational) research advances in the fields of "Annotation and prediction of structural/functional impacts of coding SNPs" and "SNPs and Personal Genomics: GWAS, populations and phylogenetic analysis". The SNP-SIG is a venue for the development of a research network of scientists, necessary for facilitating the exchange of ideas and establishing new collaborations. The 2013 SNP-SIG attracted over 100 participants, with seven research talks and five presentations from the leading scientists in the field.

The SIG topics covered in this proceedings issue address: the SNP annotation [5] from functional [6,7] and structural [8,9] perspectives, the prediction of pharmocogenomic variants [10] and new drug targets [11], the visualization of transcriptome genetic variants [12], and human population models to predict the rate of private variants [13].

[The complete program of SNP-SIG meeting 2013 with presentation and poster abstracts is available at http://snpsig.biofold.org/2013/docs/snp-sig-2013-programme.pdf].

## Further developments

The SNP-SIG is undergoing some changes, which will further promote our efforts in genome interpretation. SNP-SIG will change its name to VarI-SIG (Variant Interpretation Special Interest Group) to reach out to scientists investigating all the different types of genetic variants. We are currently working on the organization of VarI-SIG meeting (July 12, 2014) that will be held in the context of the ISMB 2014 (Boston, MA). Further information about the meeting is available on our web site (http://varisig.biofold.org). Additionally, we encourage the interested readers to join our effort to establish the VarI-COSI - Variant Interpretation Community of Special Interest - a hub for variation research-related year-round activity. VarI-COSI will be aimed at sharing relevant information, discussing ideas, and providing networks of training and support. We are in the initial stages of setting up our COSI presence on the web and look forward to input and participation from the variation interpretation community.

## Competing interests

The authors declare they have no conflict of interests in relation to this SNP-SIG issue article.

## Authors' contributions

YB and EC wrote the manuscript. Both authors read and approved the manuscript.
